# Root Filling. No. 2

**Published:** 1867-10

**Authors:** A. B. Brookins


### ARTICLE II.

### *Root Filling. No.* 2.

By A. B. Brookins, D.D.S.

The "so-called" conservative treatment having already received more consideration than it deserves, notwithstanding the prominence given it by certain reputable practitioners, we will now proceed to see what good there is in the rational system.

Different operators embrace different methods, not only in the selection of devitalizing agents, but in the preparation of the canals; some use hooked, others barbed instruments, while their patients are under the influence of gas; others dispense with the gas and with a slight of hand thrust an armed stiletto through the pulsating commissure, on through the canal stopping only at the bottom of the alveolar recess. Others again use the more humane means, arsenious acid—the *sine qua non.*

I need not mention the occasional use of the potential and actual cautery, which, however, should never leave the hand of the operating surgeon—cobalt, carbolic acid, &c., have had their advocates, but cannot be relied upon.

I am in possession of no peculiar or specific way of applying the acid further than to see that the medicine is in contact with the part to be devitalized, and do not trust it to be absorbed through a layer of dentine—the nerve should be exposed as much as possible without wounding the vascular structure; should this occur the hemorrhage should at once be arrested by the application of creosote, applied on a dossil of cotton, or better on a piece of bibulous paper. The tincture of camphor will do just as well, and be more acceptable to the taste of the patient. A preparation composed of arsenious acid, sulphate of morphia and creosote sufficient to saturate the compound, is a common devitalizing agent. A small quantity of the medicine is placed immediately on the exposed nerve, and covered loosely with cotton, saturated with sandarach varnish.

The use of this stopping has been objected to on account of the supposed solubility of the acid in the varnish, and the diffusion of its effects along the canal, destroying its lining membrane, &c. I have not found this to be the case, but like everything else, it must be done with care, and in no haste. A more eligible one I think cannot be used; wax and Hill's stopping being too hard, not unfrequently produce pain, and you are compelled to do your work over again. The medicine is allowed to remain from 12 to 24 hours, after which it is taken away, and the devitalized nerve removed as far as the foramen or mouth of the dental canal, and should there be no inflammation remaining, the gold can be at once introduced, though I prefer waiting 24 to 36 hours, lest a secondary inflammation developes itself when too late to give it successful treatment. I prefer introducing a roll of cotton of sufficient size and length, the end saturated with creosote water, as far as the foramen, taking care not to pass beyond the apex—the crown should always be kept closely filled with sandarach, wax or Hill's stopping. *The keeping out of oral fluids from the canal is at all times essential.*

Should much inflammation remain, undiluted creosote should be repeated every day till all soreness is removed. But suppose the patient applies with a devitalized organ—suppurating from the action of natural causes, that is to say, as a sequence of inflammation arising from the presence of non-destroying medical agents, or if you please from injudicious treatment.

There are several things to be taken into consideration before we can institute any course of treatment with benefit to our patients, or with credit to ourselves. What is the general health of the patient? Is he anæmic? Increase his hematin. Is there struma or a cachectic diathesis? Let your general remedies be the adjuvants of your special course. Does the patient urge penury? Divide the profits with him. "Turn not away your favor from any poor man and the Lord will not turn his face away from thee." But we find different local conditions obtaining as well as general in different cases—from congestion to suppuration—from acute to sub-acute inflammation. The affection confined to the walls of the canal, including the lining membrane; to the tubular organization producing the congestion first observed by Profs. Harris aad Johnston of Baltimore; to the soft parts of the alveolo-dental recesses, to the Crusta Petrosa and contiguous structures, or in the dental nerve (in the canal) with or without dental fistulæ, abscess, or adventitious growths, all of which may result from natural causes unassisted by artificial interference though more frequently from an unpardonable practice among some dentists.

Simple inflammation when confined to the membrane of the canal, or its preceding stage, congestion after the removal of extraneous matters, also pericementitis or any unclassified subacute inflammation outside of the dental canal, when it has not taken on the suppurative condition, may be treated with safety and success by cold water thrown along the canal to its apex. Two or three currents may be thrown at a time and repeated every 8 or 10 hours, keeping the crown well stopped with a temporary filling ad interim. In the suppurative stage we should be careful how we use cold water or in any way change the temperature ; the evidence of reputable dental pathologists to the contrary notwithstanding. I object to its use for the same reason the hospital surgeon, in his wards, rejects it from his suppurating surfaces. My friend Dr. Balderston of Baltimore, favors its adoption and says:—

"I am sure a method of treatment is arrived at so perfect that any tooth may be restored to health and filled without any danger of abscess. But it is the most careful and tedious operation the dentist has to encounter.

"If the patient calls with the nerve already devitalized and probably abscess, by cleansing the cavity and removing the debris in the canal with a suitable broach and daily syringing with *cold water*, reapplying cotton to keep out foreign matters from the crown—the abscess soon cures in from ten to fifty days, when with floss silk we fill the canal, and with tin the crown, to remain as a temporary filling for two weeks or longer. Should there be at any time signs of uneasiness in the tooth after the temporary filling has been introduced, the patient is directed to call at once and have it removed and previous treatment renewed ; if allowed to remain it will form abscess."

After a trial of the creosote treatment on suppurating surfaces along the canal, in dental fistulæ and abscesses and also in pericementitis and in over forty cases, all restored to health and filled in from four to ten days, in one fourth of which the original form was restored with solid gold, thus subjecting them to additional liability to subsequent inflammation had any morbid disposition remained, I do not feel disposed to adopt one apparently so incongruous; certainly not if it takes fifty days to do the work otherwise accomplished in ten.

The following is the method of treatment I have adopted, not differing, in general from the course pursued by Professor Gorgas of the Baltimore College of Dental Surgery, except in a few points as will be suggested as we proceed.

At first sitting of the patient, granting the dental nerve has been devitalized and its commissure removed from the crown, an untempered instrument prepared for the individual case, of proper size and having a hook sharp at the point so as to catch readily the object you wish to extract, feeling satisfied with what it brings away, and continuing with unruffled patience and unwearied hand till we arrive at the dental foramen. Do not be in a hurry nor fatigue the patient; if he wearies, you should introduce a little creasote water on cotton as far as you have reached, stop up the crown and arrange for another engagement. There are three points right here that claim special consideration, either of which if lost sight of will protract your labors or result in failure.

1st. Do not let the points of your instrument be in such relief as to tear the dental membrane, which it must be remembered is exceedingly delicate, continuous with the alveolar dental periosteum, not only adherent to the parieties of the dental canal, but sending off linings to the dental tubuli of Kolliker and forming cul de sacs in the "calcigerous" cells of Huxley or the "enamel" cells of Johnston. Within the hundreds of thousands of these tubuli are nerves, and whenever we find nerves, we must necessarily have blood-vessels, though the blood may have neither hæmatin nor fibrin and only the plastic elements of nutrition, the plasmatic fluid of some of the German Physiologists necessary to perpetuate the vitality of the parts supplied.

2nd. Do not allow any disorganized nerve or other detached fragments to remain in the vicinity of the foramen or along the parieties of the canal expecting them to come away in subsequent treatment.

3rd. By all and every means suffer not your instrument to pass beyond the mouth of the canal other than for the purpose of ascertaining the length of the canal, for which should be used a most delicate measure having the points turned at a right angle, which is very short and as it passes out a peculiar "expression" is given which is not easily misunderstood. But there is another more diagnostic and emphatic: the angle of the instrument readily embraces the apex and requires a gentle force to extract it, whilst in this embrace the distance can be taken on the shaft of the instrument which is to guide you in your subsequent treatment and in the introduction of the gold.

After the canal has been properly cleansed from foreign matters creosote is applied to the mouth of the canal on a piece of cotton rolled out, having such diameter as to be readily admitted, leaving one end outside for convenience of extraction, and the cavity in the crown so carefully stopped that oral fluids cannot permeate it. I prefer the sea island cotton to floss silk, the latter being too elastic and apt to be withdrawn with the instrument and for the same reason is introduced with less facility.

This dressing is allowed to remain from six to twenty-four hours, the former if treating abscess, fistula, &c., when much suppuration is going on, when it is removed, the canal made dry, and the treatment repeated, until all inflammation passes away and no sensibility upon percussion remains.

I now feel at liberty to proceed with my work of introducing gold along the canal to the foramen, and the same caution becomes equally important here as in the preparation of the organ, not to let any spiculæ of gold get *beyond* the apex. I see no necessity as some recommend, of allowing a pellet of cotton containing creosote to precede the gold, but on the contrary is, I doubt not, the cause of many failures. After the canal is filled I do not proceed at once to fill the crown and perhaps restore the surface or surfaces and angles, preferring to give the organ a little rest, after which the crown is properly prepared for the reception of the permanent filling, and following Dr. Atkinson who is always good authority, all difficult cavities are reduced to simple ones.

We are told it is prudent but not necessary to give the organ rest after this manipulation. I declare it both prudent and necessary, for however well the primary affection may have been treated, a secondary may be developed and all our care will bring us no reward. Right here I will say a word with regard to what I deem the most expeditious as well as successful way of introducing the gold along the canal. In the first place let your instrument be nearly of the size of the diameter of the canal and untempered so as to adapt itself to any change of direction it may assume other than direct angles, the point having a positive plane surface to force the gold directly to the mouth of the canal; tear off from the strip folded from No. 4 or 6, just the quantity as will proceed along the walls without condensing before it reaches the apex; your measurement will guide you; after this is well condensed another piece is advanced in the same way till the canal is filled. The tooth should be supported by gutta percha when the crown is being built out, or any of its surfaces. It should be used in all cases of restoration of the crown whether the root has been the subject of treatment or not.

But suppose the patient consults you for a tumor in the dome of the arch extending from the foramen incisivum backwards along the palatine suture, giving through its attenuated walls evidence of its purulent character. Taken together with the history of the case, there is no difficulty in making a correct diagnosis and finding that one or more of the incisors has been the subject of an injudicious "plug;" that the nerve was devitalized in the usual way; and receiving the stereotyped response "the doctor gave me some medicine (?) to rub on my gum if it got sore, though he assured me I would not need it, and the result has been that I have suffered agony for days and weeks." The "plug" is at once removed and the canal cleared of barriers to the free egress of pus; tepid water is then injected up the canal taking care not to throw it into the already distended sac, when the pus will freely follow the escape of the water, and the tumor relieve itself through the elasticity of its walls. A roll of cotton, the end of which is saturated with creosote is then passed up to the apex and allowed to remain; the cavity in the crown is also filled with cotton to keep out foreign matters. At the next sitting I inject the tumor with creosote and pursue the same treatment as before. It is not well to inject more than once in 24 or 36 hours, and I have never found it necessary to inject beyond the second or third time.

Again, suppose we find in the oral vestibule a hanging pocket presenting one or more openings through which passes, upon pressure, a cheesy granulated sero-purulent secretion? I have just dismissed a case of this description. The patient a young lady, aet. 18 the daughter of an eminent clergyman, was the subject of *maltreatment* 12 months since; for weeks she suffered agony till the pus discharged through the mucous membrane in the vestibule over the buccal roots of the first superior left molar; an adventitious growth had formed to half the size and shape of an almond, having several fistulous openings, and upon pressure discharged freely a consistent granulated pus, which afterwards became more fluid.

The most expeditious and advisable method of treatment for these compound cases is

1. Remove all obstructions to a free transmission of pus through the dental canal.

2. Clip away the adventitious pockets with the scissors taking care not to extend the sac with the forceps used in the operation; if you do, you will be liable to cut away the mucous membrane. The slight hemorrhage is easily controlled by the application of one of the solutions of the salts of iron.

The further treatment will be essentially the same as that instituted above in the case of abscess of the arch, excepting that the creosote may be thrown through the dental canal along the walls of the fistula, immediately after the excision of the *cul de sac.*

It has been proposed to fill the canal with gold as soon as sensibility is removed from around the root of the tooth, which is indicated by percussion, and treating the fistulous opening externally, or leaving it to cure itself; for nature to repair her own solutions of continuity. This is objectionable because of the universal disposition obtaining in fistulous ducts, to heal from the superficies to the bottom, unless you control the granulations. Now suppose you have filled the canal with care and credit so far as the operation *per se* is concerned, and union by second intention is effected in the mucous and even in the submucous tissue, you have then admirably succeeded in covering up with inflammable structures a slumbering fire, an explosive volcano with mutterings and pains longing to be delivered.

I had proposed to speak of two other conditions we not unfrequently find, but this paper is already too long, and I would prefer learning from another far more competent to instruct, the treatment adapted to their pathology.

